# Advances and Clinical Applications of Anterior Segment Imaging Techniques

**DOI:** 10.1155/2016/8529406

**Published:** 2016-12-29

**Authors:** Sang Beom Han, Jodhbir S. Mehta, Yu-Chi Liu, Karim Mohamed-Noriega

**Affiliations:** ^1^Department of Ophthalmology, Kangwon National University Hospital, Seoul, Republic of Korea; ^2^Singapore National Eye Centre, Singapore; ^3^Singapore Eye Research Institute, Singapore; ^4^Department of Ophthalmology, Yong Loo Lin School of Medicine, National University of Singapore, Singapore; ^5^Department of Ophthalmology, Autonomous University of Nuevo Leon, Monterrey, NL, Mexico

With the rapid development of computer science and technologies in recent years, there has been dramatic advance in anterior segment imaging.

As we have mentioned in the call for papers for this special issue, manuscripts are covering the topics of anterior segment imaging techniques including anterior segment optical coherence tomography (OCT), specular microscopy, corneal topography, confocal microscopy, and ultrasound biomicroscopy (UBM). These techniques have enabled precise visualization and objective assessments of anterior segment structures; thus, these devices have become essential tools for better diagnosis and treatment of anterior segment diseases including corneal disorders, cataract, glaucoma, and even disorders of the lacrimal system. Future development of novel modalities, that is, en face OCT or ultrahigh-resolution OCT, is expected to allow more detailed visualization at a microscopic level, which would provide even more understanding of anterior segment pathology at a cellular level. In this special issue, the authors contributed 15 original articles and 4 review papers regarding technologies of the anterior segment imaging and clinical application of the imaging devices.

The authors have contributed the results of their original researches on various topics on anterior segment imaging: (1) agreement between gonioscopic examination and swept source OCT imaging; (2) preliminary outcomes of accelerated corneal collagen cross-linking using topography-guided ultraviolet-A energy emission; (3) anterior segment changes before and following laser peripheral iridotomy of iris bombe in patients with uveitic secondary glaucoma; (4) anterior segment measurements with swept source OCT before and after ab interno trabeculotomy; (5) effects of a new type implantable collamer lens implantation on vision-related daily activities; (6) corneal epithelial remodeling after transepithelial photorefractive keratectomy for myopia; (7) measurement of anterior chamber volume in cataract patients using swept-source OCT; (8) biomechanical findings in contact lens-induced corneal warpage; (9) quantitative analysis of lens nuclear density using OCT with a liquid optics interface; (10) rotating Scheimpflug imaging indices in different grades of keratoconus; (11) evaluation of outflow structures in vivo after the phacocanaloplasty; (12) evaluation of corneal epithelial thickness in asymmetric keratoconic eyes and normal eyes using Fourier domain OCT; (13) central corneal thickness measured using spectral-domain OCT and associations with ocular and systemic parameters; (14) anterior segment changes in pseudophakic eyes after pars plana vitrectomy with silicone oil or gas tamponade using UBM imaging; and (15) UBM comparison of ab interno and ab externo intraocular lens scleral fixation.

This special issue also includes review articles on the following topics: (1) application of Scheimpflug imaging in glaucoma management; (2) anterior segment imaging in ocular surface squamous neoplasia; (3) applications of anterior segment OCT in cornea and ocular surface diseases; and (4) the role of anterior segment OCT and UBM in corneal and conjunctival tumors.

We hope these articles will provide readers with useful information on anterior segment imaging and new ideas for researches on related topics.

